# Acadesine for patients with relapsed/refractory chronic lymphocytic leukemia (CLL): a multicenter phase I/II study

**DOI:** 10.1007/s00280-012-2033-5

**Published:** 2012-12-11

**Authors:** Eric Van Den Neste, Bruno Cazin, Ann Janssens, Eva González-Barca, María José Terol, Vincent Levy, Jaime Pérez de Oteyza, Pierre Zachee, Andrew Saunders, Mercè de Frias, Clara Campàs

**Affiliations:** 1 Hematology Department, Cliniques universitaires UCL Saint-Luc, Brussels, Belgium; 2 Service maladies du sang, Le Centre Hospitalier Régional Universitaire de Lille, Lille Cédex, France; 3 Department of Hematology, University Hospitals Leuven, Leuven, Belgium; 4 Servicio de Hematología Clínica, Institut Català d’Oncologia, L’Hospitalet de Llobregat, Spain; 5 Departamento de Onco-Hematología, Hospital Clínico Universitario de Valencia, Valencia, Spain; 6 Laboratoire d’Hématologie, Pôle Hématologie-Oncologie Hôpital Avicenne, Bobigny Cedex, France; 7 Servicio de Onco-Hematología, Hospital Madrid Norte-Sanchinarro, Madrid, Spain; 8 Department of Hematology/Oncology, ZNA Stuivenberg, Antwerp, Belgium; 9 Linden Oncology Ltd, Edinburgh, UK; 10 Advancell Therapeutics, Advancell-Advanced In Vitro Cell Technologies S.A., Barcelona, Spain

**Keywords:** Acadesine, Relapsed-refractory CLL, Apoptosis, Phase I/III trials, Leukemias and lymphomas

## Abstract

**Purpose:**

Acadesine has shown in vitro to selectively induce apoptosis in B cells from chronic lymphocytic leukemia (CLL) patients. We conducted a phase I/II open-label clinical study, to determine the safety and tolerability of acadesine given intravenously as a 4-h infusion to CLL patients.

**Methods:**

Patient population included CLL patients with relapsed/refractory disease who had received one or more prior lines of treatment including either a fludarabine or an alkylator-based regimen. Twenty-four patients were included: eighteen in Part I treated at single doses of 50–315 mg/kg, and six in Part II, three with two doses at 210 mg/kg and three with five doses at 210 mg/kg.

**Results:**

A manageable and predictable safety profile was demonstrated for acadesine at single doses between 50 and 210 mg/kg; 210 mg/kg was the maximum tolerated dose (MTD) and optimal biological dose (OBD). Grade ≥2 hyperuricemia occurred commonly but was not clinically significant and resolved with the administration of prophylactic allopurinol. Other adverse events included transient anemia and/or thrombocytopenia (not clinically significant), renal impairment, and transient infusion-related hypotension (clinically significant). Trends of efficacy such as a reduction of peripheral CLL cells and reduction in lymphadenopathy were observed; however, the results were variable due to the small population and the range of doses tested.

**Conclusions:**

A MTD of 210 mg/kg was established with single acadesine dose. Multiple dose administrations at the OBD were tested with an acceptable safety profile, showing that acadesine might be a valuable agent for the treatment of relapsed/refractory CLL patients.

## Introduction

B-cell chronic lymphocytic leukemia (CLL) is the most frequent type of leukemia in the Western world, affecting mainly the elderly [1]. CLL follows an extremely variable clinical course, with overall survival times ranging from months to decades. The symptoms and signs of the disease arise from a clonal excess of B cells caused mainly by defects that prevent programmed cell death (apoptosis) [2].

Available treatments for CLL generally induce remission, although nearly all patients relapse and CLL remains an incurable disease [1, 3]. Fludarabine-based regimens have significantly improved the efficacy of CLL treatment, and recently, a large randomized trial showed that the addition of the anti-CD20 monoclonal antibody rituximab to fludarabine and cyclophosphamide (FC) improved progression-free survival (PFS) and overall survival (OS) in treatment-naive patients (as first-line therapy) and PFS in relapsed patients [4–6]. However, current chemotherapy is not selective for CLL cells, and attendant T-cell apoptosis leads to adverse reactions, including severe and prolonged immunosuppression. Conventional treatment of CLL is also associated with temporary myelosuppression with a concomitant increased risk of infection, anemia, and bleeding/bruising [7, 8]. Almost all patients relapse or become resistant to existing therapies, especially those with genomic alterations such as deletion 17p and/or mutations in TP53. Both have been shown to predict for poor response to chemotherapy and to be associated with poor OS [9, 10].

Acadesine (Acadra^®^), 5-aminoimidazole-4-carboxamide-1-d-ribofuranoside or AICA-riboside, is a water soluble nucleoside with a different mechanism of action compared to currently approved nucleoside analogs (e.g., fludarabine) [11]. When added to cell cultures or administered to animals or humans, acadesine is phosphorylated to its ribotide, AICA-ribotide (ZMP) [12, 13]. ZMP is a natural endogenous intermediate in the *de novo* purine nucleotide biosynthesis. Acadesine induces apoptosis of CLL cells cultured ex vivo in a dose-dependent manner over the concentration range 50 μM to 1 mM, and with an IC50 of 380 ± 60 μM [14, 15]. Incorporation of acadesine into the cell and its subsequent phosphorylation to ZMP are necessary to induce apoptosis. Moreover, acadesine is not reliant on the p53 tumor suppressor or ataxia telangiectasia mutated (ATM) proteins to drive apoptosis, and hence, the absence/loss of function of these proteins in patients with CLL should not affect the activity of acadesine. This is an important issue because alterations of either p53 or ATM are related to resistance to chemotherapy in CLL [9, 16]. Also, no significant differences in acadesine-induced apoptosis have been observed in ex vivo analysis between unmutated and mutated CLL samples and either between ZAP70 positive and ZAP70 negative cases [17]. Ex vivo, B cells were found to be much more sensitive to acadesine-induced apoptosis than T cells. Given that fludarabine causes both B- and T-cell apoptosis [7], the latter leading to the common and severe complication of immunosuppression, the B cell targeted mode of action of acadesine could provide a safety benefit over other commonly used cytotoxic chemotherapeutic regimens and a valuable treatment alternative to certain groups of patients with CLL who are refractory to other treatments.

## Patients and methods

This phase I/II study was approved by the competent institutional review board and conducted in accordance with the International Conference on Harmonization Good Clinical Practice guidelines (ClinicalTrials.gov identifier: NCT00559624).

### Patients

A total of 24 patients were enrolled in the study. All of them provided written informed consent, prior to any study related procedure not part of the patient’s normal medical care. Eligible patients had a diagnosis of CLL according to National Cancer Institute (NCI) Working Group Criteria [18], with refractory or relapsed disease, Eastern Cooperative Oncology Group (ECOG) Performance Status ≤2, and a life expectancy of at least 3 months. They had received one or more prior lines of treatment which must have included either a fludarabine- or cladribine-based regimen or an alkylator-based regimen. Refractoriness was defined as any patient who had failed to achieve a complete response (CR), nodular partial response (nPR), or partial response (PR) according to the NCI Working Group Guidelines for CLL [18]. Fludarabine refractoriness also included patients who achieved a CR, nPR, or PR of ≤6 months duration. Other inclusion criteria were adequate renal function, defined by serum creatinine ≤1.5 × upper limit of normal (ULN) and a calculated creatinine clearance of ≥60 mL/min.

### Treatment plan

This was a Phase I/II, open-label, study to evaluate an escalating dose and number of doses of acadesine in patients with CLL. The primary endpoint of the study was to demonstrate the safety and tolerability of acadesine in CLL patients, and the secondary endpoints were to determine the pharmacokinetics (PK) of acadesine and its metabolite, ZMP, and to determine the optimal biological dose (OBD) of acadesine in patients with CLL. OBD for single-dose administration of acadesine was considered as the dose enabling a plasma concentration in the range of that causing apoptosis in in vitro models determined from the PK and safety data, and was defined as the dose below that at which dose escalation is stopped in Part I of the study and this dose will be the starting dose used in Part II of the study which will assess repeat dosing with acadesine.

Patients were enrolled in the study in cohorts of three patients or more, depending upon the occurrence of dose-limiting toxicities (DLTs). Day 1 dosing was staggered between all patients in each cohort by a minimum of 48 h. In Part I, patients received a single dose of acadesine on Day 1. In Part II, patients received up to 5 doses of acadesine over a period of up to 15 days starting on Day 1. The starting dose for Part I was 50 mg/kg given as a 4 h (±30 min) intravenous (iv) infusion on Day 1 only. Patients were assessed for safety, PK, and pharmacodynamics (PD) for up to 3 weeks after dosing (to Day 22). Investigators were advised to treat patients with prophylactic allopurinol to prevent hyperuricemia in all patients in Part I from Cohort 2 onward; additionally, a specific dose and duration of treatment of allopurinol was specified for Part II. The decision to escalate to the next dose in a separate cohort of patients was based on the assessment of safety, including any DLTs, PK modeling of exposure to ZMP, and PD response data, where available, by the independent Data Monitoring Board (DMB).

Dose escalation in Part I of the study followed a modified Fibonacci dose escalation design, with 100 % dose escalations allowed until a confirmed grade 2 toxicity (as defined by NCI-Common Terminology Criteria for Adverse Events (CTCAE) version 3.0 for all toxicities except anemia and thrombocytopenia where CTCAE version 2.0 adapted for leukemia studies was used) considered related to treatment occurred. Once this occurred, future dose escalations were incremental (67, 50, 40, 33 %, etc.). In Part II, patients were treated with 2 or 5 consecutive doses at the MTD/OBD identified in Part I of the study.

### Assessments

#### Safety

Incidence, causality, and severity of adverse events (AE) and serious adverse events (SAE), local tolerability, changes in laboratory values (including liver enzymes, blood glucose and uric acid) and vital signs were assessed. They were assessed for their relationship to acadesine and classified for severity according to the CTCAE v3.0 for all events except anemia and thrombocytopenia which were assessed according to CTCAE v2.0 (which uses % changes relative to study baseline/entry).

#### Pharmacokinetics

In Part I of the study, blood samples for PK analysis (for both acadesine and its metabolite ZMP) were taken pre-dose and 0, 30, 60 min, 2, 6, 20, 72, 96, and 168 h, 14 and 21 days post-dose in all cohorts. In Part II, PK samples were also taken at pre-dose and 0 min and 20 h post-dose for any interim doses, and 72, 96, and 168 h and 14 and 21 days after completion of the last dose for each patient. Acadesine and ZMP concentrations were determined by a validated bioanalytical HPLC–MS/MS method, with lower limits of quantification (LLOQ) set at 20 ng/mL and 150 ng/mL for acadesine in human plasma and ZMP in whole blood, respectively. A noncompartmental PK analysis of the acadesine and ZMP concentrations was undertaken using the WinNonlin^®^ software, Professional Version 5.3 (Pharsight Corporation, Mountain View, CA).

#### Anti-leukemic activity

Although no International Working Group (IWG) disease response assessments were included in the protocol, bi-dimensional nodal area (sum of right neck, left neck, right axillary, left axillary, right inguinal and left inguinal nodes) and uni-dimensional lymph nodes (liver and spleen length) were measured.

#### Pharmacodynamics

The PD effect of acadesine in patients with CLL was assessed by B-cell and T-cell counts in peripheral blood. Samples were taken at pre-dose and 20, 72, 168 h, and 21 days post-dose in all cohorts. In Part II, PD samples were also taken at pre-dose and 20 h post-dose for any interim doses, and 72, 168 h and 21 days after completion of the last dose for each patient.

## Results

### Patient characteristics

From January 2008 to January 2011, 24 CLL patients with refractory or relapsed disease were enrolled onto this phase I/II clinical trial. The pretreatment characteristics are listed in Table 1. Patients were between 56 and 79 years old, all diagnosed with CLL between 4 and 21 years (inclusive) prior to study entry. The majority were males (15 patients, 63 %) and had received between 1 and 13 previous lines of therapy (mean 2.7). All patients had an ECOG score of ≤2 and had received either a fludarabine- or cladribine-based regimen or an alkylator-based regimen, in accordance with the eligibility criteria. Individual Rai and Binet staging and relapsed/refractory status at screening are displayed in Table 2.

**Table 1:** Patient demographic and baseline clinical characteristics

	PART I—single dose						PART II—multiple doses		
Dose	50	83.5	139.5	210	315	Overall part I	2 doses at 210	5 doses at 210	Overall part II
Patients	(*n* = 6)	(*n* = 3)	(*n* = 3)	(*n* = 3)	(*n* = 3)	(*n* = 18)	(*n* = 3)	(*n* = 3)	(*n* = 6)
*Age*									
Mean	69.2	66.0	67.0	67.0	60.0	66.4	69.0	66.7	67.8
Range	60–79	61–72	63–70	56–76	58–63	56–79	63–76	56–77	56–77
*Gender (number of males, %)*	2 (33 %)	2 (67 %)	2 (67 %)	2 (67 %)	3 (100 %)	11 (61 %)	2 (67 %)	2 (67 %)	4 (67 %)
*Duration of disease (years)*									
Mean	7.6	15.0	12.7	10.4	11.0	10.7	10.7	10.8	10.7
Range	4–14	14–17	5–21	7–13	7–14	4–21	7–13	6–16	6–16
*Performance status*									
0	3	2	2	3	1	11 (61 %)	0	2	2 (33 %)
1	2	1	1	0	2	6 (33 %)	2	1	3 (50 %)
2	1	0	0	0	0	1 (6 %)	1	0	1 (17 %)
*Rai stage III*–*IV*	1	1	2	1	1	6 (33 %)	1	0	1 (17 %)
*Baseline FISH analysis*									
Del 13q14	4 (67 %)	3 (100 %)	1 (33 %)	0	1 (33 %)	9 (50 %)	1 (33 %)	2 (67 %)	3 (50 %)
Del 11q22–23	1 (17 %)	1 (33 %)	1 (33 %)	0	1 (33 %)	4 (22 %)	1 (33 %)	1 (33 %)	2 (33 %)
Trisomy 12	1 (17 %)	0	0	0	0	1 (6 %)	2 (67 %)	1 (33 %)	3 (50 %)
Del 17p	1 (17 %)	1 (33 %)	0	0	0	2 (11 %)	0	0	0
*Number of patients with number of prior therapies* >*1*	5	2	2	3	3	15 (83 %)	2	1	3 (50 %)
Range	1–4	1–13	1–2	2	2–6	1–13	1–7	1–4	1–7
*Absolute lymphocyte count (10* ^*9*^ */L)*									
Mean	40.5	30.1	67.2	70.2	31.2	46.6	132.3	16.5	74.5
Range	15.7–85.0	11.6–53.3	38.4–90.5	15.7–106.0	1.0–68.6	1.0–106.0	50.0–229.4	9.8–29.3	9.8–229.4
*Hemoglobin (g/L)*									
Mean	120.3	123.3	129.0	103.7	114.7	118.5	106.0	130.7	118.3
Range	110–136	114–129	107–144	103–105	99–123	99–144	9–115	116–142	99–142

**Table 2:** Individual Rai–Binet stage and relapsed/refractory status at screening

Cohort	Dose (mg/kg)	Patient	Rai stage	Binet stage	Relapsed or refractory^a^
*Part I*					
1	50	1	1	A	Relapsed
		2	4	C	Relapsed
		3	0	A	Refractory
		4	1	A	Refractory
		5	2	A	Refractory
		6	1	A	Relapsed
2	83.5	7	0	A	Relapsed
		8	1	B	Relapsed
		9	4	C	Relapsed
3	139.5	10	4	C	Relapsed
		11	4	C	Refractory
		12	1	A	Relapsed
4	210	13	4	C	Relapsed
		14	2	A	Relapsed
		15	2	A	Relapsed
5	315	16	1	B	Relapsed
		17	4	C	Relapsed
		19	NA	A	Relapsed
*Part II*					
6	2 × 210	20	1	A	Relapsed
		22	3	B	Relapsed
		23	2	B	Relapsed
7	5 × 210	24	NA^b^	NA^b^	Relapsed
		25	2	B	Relapsed
		26	2	B	Relapsed

*NA* no data available

^a^Refractoriness is defined as any patient who has failed to achieve a complete response (CR), nodular partial response (nPR) or partial response (PR) according to the National Cancer Institute Criteria (NCI) working guidelines for CLL. Fludarabine refractoriness also includes patients who achieved a CR, nPR or PR of ≤6-month duration

^b^Only Day 22 post-treatment available: Rai stage 0, Binet stage A

### Treatment

In Part I of the study, patients received a single dose of acadesine on Day 1 at escalating doses: 50 mg/kg (6 patients), 83.5 mg/kg (3 patients), 139.5 mg/kg (3 patients), 210 mg/kg (3 patients), and 315 mg/kg (3 patients). At the 315 mg/kg dose level, 2 of the 3 patients experienced a DLT. Also, PK data indicate that at 315 mg/kg, a plateau in acadesine conversion to ZMP was reached. Therefore, 210 mg/kg was designated per protocol MTD and OBD for single acadesine administration based on the decision of the DMB. The dose administered in Part II (210 mg/kg) was based on the results of Part I. In view of the possible risk of acadesine-related renal adverse events or laboratory evidence of creatinine elevation, either as part of a clinical tumor lysis syndrome or occurring as isolated renal impairment, acadesine in Part II Cohort 1 was to be administered with a minimum of a 72-h interval between each dose. Thus, 3 patients received 2 doses (on Days 1 and 4) and 3 patients received 5 consecutive doses (on Days 1, 4, 8, 11, and 15).

### Safety and tolerability results

Acadesine infusions were well tolerated. No acadesine-related deaths occurred in the study, and no patients withdrew from the study due to AEs (all causality, either related or not related to the study drug), in either the single or repeated dose part of the study. In Part I, a total of 78 AEs were reported in 15 (83 %) patients, and approximately half of all AEs were reported as drug-related (42 AEs, 54 %. See Table 3). Six SAEs were reported in 2 patients in the 50 mg/kg (3 SAEs) and in 2 patients in the 315 mg/kg (3 SAEs) cohorts. In Part II, a total of 48 AEs were reported in 6 patients, over half of all AEs were reported as drug-related (31 AEs, 65 %. See Table 3), and there were no SAEs. Among all study patients, a single CTCAE grade 4 AE was reported (hyperuricemia) and 9 CTCAE grade 3 events (in 6 patients) were reported.

**Table 3:** Incidence of drug-related adverse events

	Part I							Part II		
mg/kg	50		83.5	139.5	210	315		2 doses at 210		5 doses at 210
AEs CTCAE grade	1–2	3–4	1–2	1–2	1–2	1–2	3–4	1–2	3–4	1–2
Number possible, probable or definitive drug-related AEs (number of patients)	11 (4)	3 (2)	5 (2)	1 (1)	4 (3)	15 (3)	3 (2)	9 (3)	1 (1)	21 (3)
*Investigations*										
Alanine aminotransferase increased	2 (1)									
Blood bilirubin increased	1 (1)					1 (1)				
Blood creatinine increased							1 (1)			3 (2)
Platelet count decreased	1 (1)	1 (1)								
*Nervous system disorders*										
Headache	1 (1)					1 (1)		1 (1)		1 (1)
Dizziness						1 (1)				1 (1)
Paresthesia	1 (1)									
Presyncope						1 (1)				
*Metabolism and nutrition disorders*										
Hyperglycemia	1 (1)	1 (1)								
Hyperkalemia			1 (1)		1 (1)					
Hyperuricemia		1 (1)								
Hypoglycemia			1 (1)							
Hypokalemia						1 (1)				
Tumor lysis syndrome							1 (1)			
*Gatrointestinal disorders*										
Diarrhea			2 (1)			1 (1)				1 (1)
Nausea						2 (2)		1 (1)		5 (2)
Gastric disorder										
Vomiting						1 (1)		1 (1)		
Abdominal pain										1 (1)
*Vascular disorders*										
Hypotension					1 (1)	1 (1)		1 (1)		
Hypertension	1 (1)									
Hematoma						1 (1)				
*Blood and lymphatic disorders*										
Anemia (or hemoglobin decreased)	1 (1)		1 (1)			3 (1)		3 (2)		6 (2)
Thrombocytopenia								2 (2)		
Neutropenia									1 (1)	
*Renal and urinary disorders*										
Renal failure acute							1 (1)			
*Other non-hematological AEs*										
Other AEs	2 (2)			1 (1)	2 (2)	1 (1)				3 (1)

Only possible, probable, or definitive drug-related AEs are shown here. In the cohorts 83.5, 139.5, 210 mg/kg in Part I, and in the cohort with 5 doses at 210 mg/kg in Part II, any CTCAE grade 3 or 4 was reported. The number of patient that reported the AEs is indicated in brackets for each event

In part I of the study, one patient at 50 mg/kg experienced hyperuricemia (CTCAE grade 4, DLT but not reported as an SAE). As a result, prophylactic allopurinol was administered to patients in the higher dose cohorts, and no further hyperuricemia AEs occurred. Two patients, one at 210 mg/kg and one at 315 mg/kg, reported acadesine-related transient hypotension, CTCAE grade 1 and 2, respectively, starting after acadesine infusion and resolved the day after acadesine dosing (Day 2) with no clinical sequelae. Only the patient treated with 315 mg/kg required treatment (a sodium chloride iv infusion) and had concomitant AEs of presyncope (vasovagal syndrome) and nausea, both CTCAE grade 1. In Part I, the most commonly reported drug-related AEs in Part I were anemia (CTCAE grade 1 or 2, reported in 3 patients, all of them with hemoglobin values below the normal range at pre-dose) and diarrhea, reported in 2 patients, of CTCAE grade 1 (see Table 3).

At 315 mg/kg, 2 out of 3 patients experienced DLTs: the first patient experienced a tumor lysis syndrome (classed as a SAE, CTCAE grade 3), which resolved within 13 days with rasburicase treatment. The second patient, diagnosed with small lymphocytic lymphoma and a large abdominal lymphomatous mass, reported renal impairment/increased creatinine (both reported as SAEs and CTCAE grade 3), which resolved quickly (returning to grade 1 within 14 days) with conservative treatment (no dialysis required).

In the repeat dose phase, no SAEs were observed. There was evidence of transient increases in creatinine after dosing in the 5 × 210 mg/kg repeat dose cohort: one DLT was reported in one patient (increased creatinine; CTCAE grade 2) and an AE was reported in another patient (increased creatinine; CTCAE grade 1), both patients having normal creatinine levels at baseline. One patient treated with 2 doses at 210 mg/kg reported acadesine-related hypotension (CTCAE grade 2), which resolved spontaneously within 24 h, without treatment and without clinical sequelae. In Part II, the most commonly reported drug-related AEs during repeat dosing were anemia (CTCAE grade 1 or 2; reported in 5 patients, 4 of them with hemoglobin values below the normal range at pre-dose), thrombocytopenia (CTCAE grade 1 or 2, reported by 2 patients, one of them with platelets pre-dose levels below the normal range), and nausea (reported by 3 patients; CTCAE grade 1 or 2).

### Pharmacodynamic and anti-leukemic activity results

There was reduction in the size of palpable lymph nodes after single and repeat dosing with acadesine, although it should be noted that formal IWG response criteria [18] assessments were not planned in this phase I/II study. In Part I, 11/18 patients showed evidence of anti-leukemic activity, in terms of decreases in B cells and/or decreases in the size of lymph nodes (decreases >20 % at any time after dosing with respect to pre-dosing values in either B-cell counts or lymph nodes size. See Table 4). In Part II, 5/6 patients showed evidence of anti-leukemic activity according to the same criteria. For example, a patient with Del13q14 and Del17p, treated at 50 mg/kg, experienced a sustained reduction in B-cell counts, which at Day 22 post-treatment was 58 % lower than at baseline. Also, one patient treated with five doses at 210 mg/kg experienced a reduction in B-cell counts up to 65.7 % on Day 8 post-baseline. This patient had lymph nodes involvement and paraneoplastic skin manifestations. The lymphocyte count has continued to drop, and 18 months after acadesine treatment, lymphocyte count is below 5,000/μL, and there are no signs of disease in lymph nodes or skin.

**Table 4:** Pharmacodynamic results

Part	Cohort	Patient number	Cytogenetic alterations^a^	B cells (cells/mm^3^)					Sum of bi-dimensional Lymph nodes (cm)			Sum of uni-dimensional liver and spleen nodes (cm)		
				Day 1 (pre-dose)	Day 2	% Variation Day 2 versus Day 1	Day 8	% Variation Day 8 versus Day 1	Day 1 pre-dose	Day 8	% Variation versus Day 1	Day 1 pre-dose	Day 8	% Variation versus Day 1
I Single dose	Cohort 1 50 mg/kg	1	Del 13q14	19,958	15,730	−21 %	22,839	14 %	2	1	−50 %	ND	ND	–
		2	Del 13q14	84,053	NA	–	77,607	−8 %	7, 5	7, 5	0 %	2	ND	−100 %
		3	NA	19,069	15,845	−17 %	18,734	−2 %	ND	ND	–	ND	ND	–
		4	Del 13q14 Del 11q22	25,274	21,856	−14 %	22,926	−9 %	3	3	0 %	ND	ND	–
		5	t 12^b^	84,466	78,811	−7 %	67,663	−20 %	ND	ND	–	ND	ND	–
		6	Del 13q14 Del 17p^c^	15,903	15,141	−5 %	8,244	−48 %	2	2	0 %	ND	ND	–
	Cohort 2 83.5 mg/kg	7	Del 13q14	56,506	43,464	−23 %	62,890	11 %	ND	ND	–	ND	ND	–
		8	Del 13q14 Del 17p^d^	11,682	14,862	27 %	17,326	48 %	3	6	100 %	ND	ND	–
		9	Del 13q14 Del 11q22	23,608	22,973	−3 %	28,419	20 %	8	12 ^g^	50 %	18	13 ^g^	−28 %
	Cohort 3 139.5 mg/kg	10	No alterations	92,967	67,814	−27 %	83,055	−11 %	6	6	0 %	4	1	−75 %
		11	Del 13q14	70,717	NA	–	82,019	16 %	ND	ND	–	5	5	0 %
		12	Del 11q22	35,073	25,140	−28 %	33,130	−6 %	ND	2	100 %	ND	ND	–
	Cohort 4 210 mg/kg	13	No alterations	94,500	98,464	4 %	103,630	10 %	4, 5	5, 5	22 %	1	1	0 %
		14	No alterations^e^	88,556	60,840	−31 %	87,782	−1 %	ND	ND	–	2	2	0 %
		15	NA	14,330	13,483	−6 %	15,163	6 %	2, 5	2	−20 %	ND	ND	–
	Cohort 5 315 mg/kg	16	No alterations	18,739	22,047	18 %	8,653	−54 %	16	11	−31 %	ND	ND	–
		17	Del 13q14 Del 11q22	62,634	61,416	−2 %	68,075	9 %	10	9	−10 %	8	7	−13 %
		19	No alterations^f^	NA	NA	–	NA	–	NA	NA	–	NA	NA	–

Part	Cohort	Patient number	Cytogenetic alterations^a^	Day 1 pre-dose	Day 2, after the last dose	%variation Day 2 after last dose versus Day 1	Day 8, after the last dose	% Variation versus Day 8 after last dose versus Day 1	Day 1 pre-dose	Day 8, after the last dose	% Variation versus Day 1	Day 1 pre-dose	Day 8, after the last dose	% Variation versus Day 1
II Multiple doses	Cohort 1 2 doses at 210 mg/kg	20	Del 13q14 t 12	110,267	78,062	−29 %	94,694	−14 %	1, 4	2	43 %	ND	ND	–
		22	No alterations	230,364	225,421	−2 %	296,661	29 %	15	16 ^g^	7 %	ND	ND	–
		23	t 12 Del 11q22	61,360	40,124	−35 %	83,364	36 %	31	31	0 %	2	2	0 %
	Cohort 2 5 doses at 210 mg/kg	24	Del 13q14	7,240	13,448	86 %	6,440	−11 %	ND	ND	–	ND	ND	–
		25	Del 13q14 Del 11q22	31,068	75,574	143 %	63,013	103 %	4	2	−50 %	ND	ND	–
		26	t 12	9,177	5,569	−39 %	3,145	−66 %	1	4, 5	350 %	1	ND	−100 %

*NA* no data available, *ND* not diseased

^a^Cytogenetic alterations (del 13q14, del 11q22, del 17p and t 12) were assessed by FISH analysis. The thresholds for alterations, defined as the percentage of nuclei carrying the studied alteration, were >5 % for del 13q14, and >10 % for del 11q22, t 12 and del 17p

^b^Del 13q14, del 11q22 and del 17p not assessed

^c^26 %

^d^34 %

^e^Del 6q probe was used in this patient, and found positive

^f^Del 11q22, t 12 not assessed

^g^Day 22 data (day 8 data not available)

Although we only had data from 3 patients per cohort (6 in cohort 1), we compared the B- and T-cell mean values at every post-treatment time with values at pre-treatment. Changes in T-cell counts were inconsistent across the cohorts and no overall significant trend was apparent. For B cells, statistically significant decreases occurred at some time points in some cohorts (see Table 4). In cohort 1, decrease in B cells at Day 2 was statistically significant using parametric (*p* = 0.012, Student *t* test) or nonparametric (*p* = 0.043, Wilcoxon signed rank test) method. Similarly, when pre-dose B cells were compared to Day 2 results in all 15 patients from the single-dose cohort where both values are available, a significant difference was found by both statistical methods (*p* = 0.038 and *p* = 0.027, respectively). This suggests a trend in decrease in B cells and tends to confirm the results from in vitro experiments, where acadesine induced apoptosis selectively in B cells [14].

### Pharmacokinetic results

**Fig. 1:**
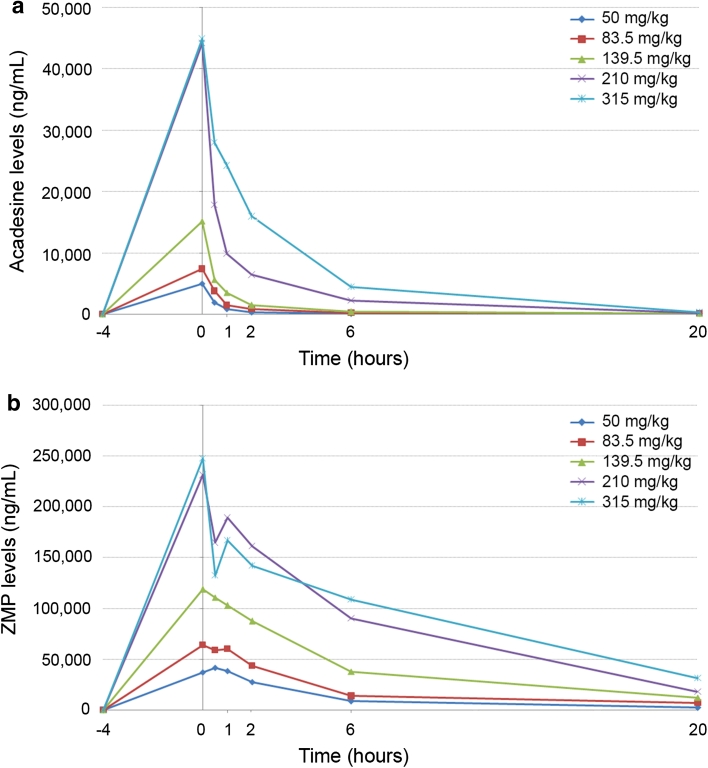
Acadesine mean plasma levels (ng/mL) (**a**) and ZMP mean whole blood levels (ng/mL) (**b**) are represented at 4 h pre-dosing (−4) and 0, 0.5, 1, 2, 6, and 20 h post-acadesine administration. For 50 mg/kg, *n* = 6, for 83.5 mg/kg, 139.5 ng/kg, and 315 ng/kg *n* = 3, and for the optimal biological dose 210 mg/kg, data from 9 patients is shown (3 patients at 210 mg/kg single dose, 3 patients at 2 × 210 mg/kg and 3 patients at 5 × 210 mg/kg). For the patients that received several acadesine administrations, only data from the first dose have been used to calculate the mean acadesine and ZMP levels

Results showed that acadesine and ZMP were rapidly distributed in the bloodstream, and acadesine was rapidly converted into ZMP. Maximum acadesine concentrations were observed at the end of the infusions and from then on concentrations started to decrease. The LLOQ for acadesine was reached between 20 h and 15 days. Maximum concentrations of ZMP were found between the end of the infusion and 30 min–1 h later due to the time elapsed between acadesine administration and its transformation into ZMP. Blood ZMP concentrations started decreasing progressively from 1 h on (after the end of the infusion), reaching the LLOQ between 5 and 22 days after administration (Fig. 1). At the OBD (210 mg/kg single dose), maximum concentration (*C*_max_) for acadesine was 38,736 ng/mL and 270,988 ng/mL for ZMP (median of the 3 patients treated in the cohort). The Area under the curve (AUC_0→24_) was 123,124 ng h/mL for acadesine and 2,066,170 ng h/mL for ZMP (median of 3 patients treated in the cohort). Considering that the sum of the *C*_max_ molar plasma concentrations of acadesine and ZMP, obtained at the end of the 4-h infusion, would be equivalent to the dose used in an in vitro cell culture, at the OBD dose (210 mg/kg single dose) target acadesine concentrations were achieved (median acadesine + ZMP concentration was 0.9 mM, higher than the 0.5 mM concentrations used to induce apoptosis in in vitro cell cultures) [14, 15]).

Time of maximum drug concentration (*T*_max_), maximum drug concentration (*C*_max_), area under the time–concentration curve from 0 to 24 h (AUC_0→24_), and half life (*t* **½**) for all cohorts are summarized in Table 5.

**Table 5:** Pharmacokinetic parameters (a) acadesine, (b) ZMP

Cohort	Dose (mg/kg)	*t*_max_ (h)	*C*_max_ (ng/mL)	AUC_(0→24h)_ (ng h/mL)	*t*_½_ (h)
*(A)*					
Part I					
1	50				
	Median	4.0	4,325	14,251	5.2
	Minimum	3.7	2,930	9,800	1.0
	Maximum	4.3	8,139	21,213	45.4
2	83.5				
	Median	4.1	6,888	24,614	35.0
	Minimum	4.1	5,148	15,986	5.2
	Maximum	4.5	10,036	32,534	79.4
3	139.5				
	Median	4.0	12,439	39,503	25.3
	Minimum	3.9	2,449	11,549	3.8
	Maximum	4.0	30,574	89,015	28.5
4	210				
	Median	4.0	38,736	123,125	16.2
	Minimum	4.0	30,431	118,235	10.2
	Maximum	4.1	64,672	240,500	35.7
5	315				
	Median	4.5	35,170	240,403	61.4
	Minimum	4.2	33,855	171,809	2.6
	Maximum	4.7	65,536	275,298	62.0
Part II					
6	2 × 210				
	Day 1				
	Median	4.1	41,851	195,556	24.6
	Minimum	4.0	7,177	26,315	14.2
	Maximum	4.4	68,366	228,532	25.8
	Day 4				
	Median	4.2	28,774	126,430	98.8
	Minimum	4.0	2,213	14,858	18.8
	Maximum	4.5	45,939	149,534	106.2
7	5 × 210				
	Day 1				
	Median	4.3	49,533	161,939	3.9
	Minimum	4.3	41,619	117,843	3.0
	Maximum	4.6	54,892	198,014	12.0
	End of treatment				
	Median	4.1	51,456	198,646	56.2
	Minimum	4.0	51,160	148,617	3.8
	Maximum	4.2	67,684	231,356	65.1
*(B)*					
Part I					
1	50				
	Median	4.3	52,101	312,131	72.3
	Minimum	3.67	7,666	20,438	0.5
	Maximum	5.03	93,198	424,582	239.9
2	83.5				
	Median	4.5	73,023	562,247	35.1
	Minimum	4.08	71,775	360,973	34.5
	Maximum	5.08	76,814	577,760	95.2
3	139.5				
	Median	4.0	114,001	1,023,992	66.4
	Minimum	4.00	94,144	701,177	17.6
	Maximum	4.58	156,663	1,343,691	91.2
4	210				
	Median	4.1	270,988	2,066,170	39.8
	Minimum	4.00	185,258	1,910,548	22.2
	Maximum	5.08	295,124	2,159,142	118.2
5	315				
	Median	4.5	243,540	2,434,242	8.1
	Minimum	4.17	157,154	1,472,810	5.7
	Maximum	4.68	342,195	2,873,273	111.7
Part II					
6	2 × 210				
	Day 1				
	Median	4.1	275,836	2,610,904	24.3
	Minimum	4.00	152,335	853,666	12.7
	Maximum	4.42	312,929	2,747,816	26.5
	Day 4				
	Median	4.7	223,390	2,081,843	81.5
	Minimum	4.00	95,310	738,586	64.4
	Maximum	5.50	229,030	2,538,271	134.1
7	5 × 210				
	Day 1				
	Median	5.7	265,536	1,833,232	16.6
	Minimum	5.33	159,539	1,056,891	11.4
	Maximum	6.33	344,686	2,866,182	45.4
	End of treatment				
	Median	4.2	188,936	1,942,209	71.3
	Minimum	4.00	108,108	870,256	16.2
	Maximum	5.08	265,657	2,152,337	98.1

*T*_*max*_ time of maximum drug concentration, *C*_*max*_ maximum drug concentration, *AUC*_*0→24*_ area under the time–concentration curve from 0 to 24 h, *t½* and half life

The PK analysis of both acadesine and ZMP demonstrates a high degree of inter-subject variability. For both cohorts in Part II, there was no important accumulation of acadesine or ZMP upon multiple dosing.

Following single-dose infusion of acadesine, there was a trend toward a plateau in ZMP levels after increasing the acadesine dose from 139.5 to 210 mg/kg. PK data at 315 mg/kg and PK modeling indicate a plateau in acadesine conversion to ZMP. Thus, the OBD determined from the PK and safety data was 210 mg/kg.

## Discussion

This study was the first administration of acadesine in cancer patients—specifically CLL. A manageable and predictable safety profile was demonstrated for acadesine in patients with CLL at single doses between 50 and 210 mg/kg. The key safety findings with regard to AEs and clinical laboratory results were asymptomatic hyperuricemia, transient renal impairment/increased creatinine, and infusion-related hypotension. Hyperuricemia has been documented in previous studies with acadesine administered to prevent ischemic reperfusion injury, and given that acadesine is metabolized to uric acid [12, 13], hyperuricemia was not unexpected in patients with CLL. Prophylactic allopurinol adopted from cohort 2 (Part I) resulted in no further hyperuricemia occurring in the study. Four patients had renal impairment during the study (two received 315 mg/kg and two 5 × 210 mg/kg). All of them recovered with appropriate management and none of them required dialysis. Two independent nephrologists reviewed data from all patients and conclude that the renal dysfunction associated with acadesine treatment exhibited functional characteristics rather than nephrotoxic features, based on the rapid reversibility and full recovery of the renal dysfunction even after several acadesine consecutive doses. They proposed renal function monitoring and prophylaxis measures that will be implemented in the following clinical trials with acadesine. Modest (grade 1 or 2), transient anemia and/or thrombocytopenia, with recovery to baseline levels occurring in most patients within last days of acadesine administration, were reported in 3/18 and 5/6 patients treated in Part I and Part II of the study, respectively. All but one these AEs were drug-related, and none of them were clinically significant. Acadesine-related transient hypotension occurred in 3 patients in this trial. The hypotension typically occurred after acadesine infusion and lasted several hours with no clinically significant sequellae. Recovery occurred either spontaneously or with conservative (iv fluid administration) management. Of note, there was no evidence of renal toxicity or hypotension in pre-clinical studies or in cardiac studies in more than 2,000 patients who were administered acadesine [19, 20].

This is the first time that the PK of acadesine has been investigated in patients with CLL. The results indicate a high degree of inter-subject variability. Several possible factors may be contributing to this observation, for example: the underlying disease, associated co-morbidities, and co-medication. The OBD, that is, the dose enabling a plasma concentration in the range of that causing apoptosis in in vitro models determined from the PK and safety data, was 210 mg/kg.

In terms of disease activity, there were no conventional IWG responses in this study; such assessments were not prospectively planned as is typical in a phase I trial. However, there was evidence of anti-leukemic activity in 16/24 patients based on a more than 20 % reduction in either B-cell count or size of palpable lymph nodes. In vitro anti-leukemic activity independent of genetic alterations has been previously described with acadesine by Pairet and collaborators [17]. Our study size is too small to draw definitive conclusions, but indications of anti-leukemic activity were seen in patients with poor prognosis genetic alterations, suggesting that acadesine might be a potential treatment option in this sub-group.

The absence of significant reduction in T-cell counts with acadesine in this study is in agreement with ex vivo data which showed B cells were more sensitive to acadesine-induced apoptosis than T cells [14]. This is in contrast with current chemotherapy agents, such as fludarabine, which cause long-lasting T- and NK-cell depression. [7]. Although early indications from this study are that acadesine does not induce T-cell apoptosis to the same extent as existing treatments, larger scale studies will be required to confirm this in the wider population.

In conclusion, this study has defined the DLT, MTD, and OBD of acadesine and also shows provisional evidence of acadesine anti-leukemic activity, with an acceptable and manageable safety profile. Assessment and monitoring of renal function is essential in patients receiving acadesine. The modest myelosuppressive profile observed with acadesine (if confirmed in larger trials) may make it either an attractive combination partner for standard of care agents used in CLL patients or a candidate for post-induction intervention. The p53-independent mechanism of action of acadesine makes it an attractive compound in patients bearing alterations of this pathway, which represent a significant group in advanced patients, and for whom few therapeutic options are available. Preliminary data also show that acadesine has in vitro toxicity toward other B-cell malignancies, such as mantle cell lymphoma (especially when combined with monoclonal antibodies [21]) and multiple myeloma [22]. Thus, acadesine could be a worth candidate to be developed in other aggressive leukemic indications in the near future.
